# The Diagnostic, Prognostic, and Therapeutic Potential of Cell-Free DNA with a Special Focus on COVID-19 and Other Viral Infections

**DOI:** 10.3390/ijms241814163

**Published:** 2023-09-15

**Authors:** Galina Hovhannisyan, Tigran Harutyunyan, Rouben Aroutiounian, Thomas Liehr

**Affiliations:** 1 Department of Genetics and Cytology, Yerevan State University, Alex Manoogian 1, Yerevan 0025, Armenia; galinahovhannisyan@ysu.am (G.H.); tigranharutyunyan@ysu.am (T.H.); genetik@ysu.am (R.A.); 2 Jena University Hospital, Friedrich Schiller University, Institute of Human Genetics, Am Klinikum 1, 07747 Jena, Germany

**Keywords:** COVID-19, cfDNA, cell death, extracellular vesicles, viral infections

## Abstract

Cell-free DNA (cfDNA) in human blood serum, urine, and other body fluids recently became a commonly used diagnostic marker associated with various pathologies. This is because cfDNA enables a much higher sensitivity than standard biochemical parameters. The presence of and/or increased level of cfDNA has been reported for various diseases, including viral infections, including COVID-19. Here, we review cfDNA in general, how it has been identified, where it can derive from, its molecular features, and mechanisms of release and clearance. General suitability of cfDNA for diagnostic questions, possible shortcomings and future directions are discussed, with a special focus on coronavirus infection.

## 1. Introduction

The recently increased interest in extracellular DNA is associated with the possibility of its application as a biomarker for general screening and monitoring of disease progression and therapy response, as well as a druggable target [1]. Cell-free DNA (cfDNA), freely circulating in the bloodstream, urine, and other fluids (or encapsulated in vesicles) may be derived from both normal and diseased cells. cfDNA is extremely dynamic and responsive, providing sensitive indicators of changes that are not detectable by standard clinical tests. It can be used as a reliable, safe, and objective tool to reflect disease progression and supplement clinical data in a particular patient, and thus, represents a new path in personalized medicine [2].

An increasing number of studies demonstrate the potential use of cfDNA as a noninvasive biomarker to determine pathologic conditions in various diseases, in particular diabetes [3], autoimmune rheumatic diseases [4], non-infectious inflammations and tumors [5], as well as brain diseases [6,7]. In addition, placenta-derived—so-called cell free fetal DNA (cffDNA)—is aggressively marketed, irrespective of its known restrictions in the frame of the “non-invasive prenatal testing” (NIPT). For a review, see [8].

Given the challenges posed by the coronavirus pandemic, in this review we focus on the potential of cfDNA as a possible marker for risk assessment during COVID-19/coronavirus and other viral infections, whereby cfDNA may be of host or of viral origin. Detection of microbial/ viral cfDNA using next generation sequencing (NGS) is an accurate approach to identify and quantify pathogens in clinical specimens. While viral DNA is indicative of acute infection, host cfDNA indicates an organism’s response to infection. This review concentrates on host cfDNA but not viral cfDNA.

Overall, a brief general overview on the discovery of cfDNA, its sources, characteristics, clearance, and potential biological significance is provided, summarizing also novel cfDNA research opportunities being applicable in vitro and in vivo, paving the way for improved cfDNA-based diagnosis, prediction, prognosis, and drug development.

## 2. Brief Overview of Cell-Free DNA

According to Grabuschnig et al. [9], the term cfDNA encompasses all kinds of extracellular DNA molecules found in serum or plasma and other body fluids, and includes genomic DNA (gDNA) and mitochondrial DNA (mtDNA), as well as DNA of bacterial or viral origin. A pathology-related increase of cfDNA level was first detected in patients with systemic lupus erythematosus in 1966 [10] and cancer patients [11] in 1977. Moreover, Leon et al. [11] found a decrease of cfDNA level after anticancer therapy. The identification of placenta-derived cfDNA in maternal blood in 1997 [12] led to the establishment of NIPT cell-free mtDNA (cf-mtDNA) was first reported in serum and plasma of patients with type 2 diabetes mellitus and healthy donors in 2000. Hereby, an A to G 3243 mutation was identified in cf-mtDNA of diabetes patients but not in a control group [13].

Elevated levels of host cfDNA in the blood (plasma or serum) have been found in various viral diseases, as well as in COVID-19. Initially, an increase in cfDNA and its prognostic role was reported in patients with acute dengue virus infection [14,15]. An increase of cfDNA level in plasma during acute Puumala hantavirus infection, as well as its positive correlation with blood leukocyte concentration and length of hospital stay was found [16]. Elevated levels of plasma cfDNA have been found in patients with Hantaan virus infection [17]. The difference between cfDNA levels was revealed in mild, intermediate, and severely affected patients with Crimean–Congo hemorrhagic fever caused by a tick-borne virus (Nairovirus) [18]. cfDNA as a marker of COVID-19 diagnostics and management is currently under study [19]. In addition to freely circulating, cfDNA attached to extracellular vesicles, as well as cfDNA as part of neutrophil extracellular traps (NETs) has been studied in viral pathologies.

While cfDNA research and application had a slow start, at present, a rapid progress is observed, due to its great potential as a minimally invasive source of diagnostically relevant information, including patient-centered diagnosis, prognosis, prediction, and monitoring [20].

### 2.1. Cell-Free DNA Concentration and Size

The cfDNA content in the bloodstream is heterogeneous and depends on different biological and physiological factors [9]. The comprehensive review of Yuwono et al. [21] summarizes results from 66 studies published between January 2000 and January 2021 on the contribution of biological (body mass index, menstruation, hypertension, circadian rhythm, psychological stress, biological sex, and age) and lifestyle factors (acute and chronic exercise, alcohol consumption, occupational hazard exposure, and smoking) to cfDNA variability [21]. The most pronounced but short-term effect on cfDNA concentration was observed for (i) physical activity, followed by (ii) alcohol consumption and menstruation having slight effects, and (iii) other factors; both an increase and decrease of cfDNA levels can be the result. Disruption of DNA release and clearance balance can also impact cfDNA levels. The cfDNA half-life in circulating blood varies from several minutes to 1–2 h [22]. Healthy individuals have low cfDNA levels as apoptotic cells together with cfDNA are rapidly cleared. In malignancies, chronic inflammation, or excessive cell death, clearance is insufficient, and cfDNA accumulates [22]. Thus, the balance of cfDNA generation and clearance may be vital for health and disease [23].

The cfDNA profile in any body fluid is generally highly complex, often consisting of DNA fragments of diverse size and origin [20]. The correspondence of cfDNA length to nucleosome size means it is of apoptotic origin [9]. Longer DNA fragments (i.e., >10 kilobase pairs (kbp)) are considered to be products of necrotic cell death. Shorter DNA fragments (<100 base pairs (bp)) are enriched with mtDNA and can also include tumor- and bacterial-derived cfDNA [22,24]. Thus, variations in concentration and size distribution of cfDNA molecules might be useful as additional parameters for monitoring different physiological conditions of the organism. The main approach for the analysis of cfDNA fragment sizes and composition is NGS. Based on such NGS studies, fragmentomics—a study of the fragmentation patterns of cfDNA at single-base resolution—was developed [25,26]. Although data on cfDNA fragment size in patients with viral infections are scarce, further developments in this direction may provide new tools for discrimination of patients by disease severity [27].

### 2.2. Sources and Biological Activity of Cell-Free DNA

cfDNA origin in body fluids is mainly associated with cell death through apoptosis, necrosis, and NETosis. It can also be formed as a result of erythroblast enucleation and chromosomal instability. Furthermore, cfDNA can be present in its free form or encapsulated in extracellular vesicles (EVs). The main manifestations of the biological activity of cfDNA are regulation of the immune system, removal of damaged DNA, and intercellular communication.

#### 2.2.1. Apoptosis, Necrosis and NETosis

Cell death results in massive release of cellular contents into the extracellular space. Increased cfDNA levels have been reported for various diseases, including viral infections, in which cell death and tissue/organ damage contribute to pathogenesis. Cell death pathways are initiated during viral attachment, entry, genome replication, and gene expression.

Apoptosis, as well as two forms of regulated necrosis—necroptosis and pyroptosis, are considered as the main ways of virus associated cell death [28,29]. cfDNA can also originate from neutrophil extracellular traps (NETs), web-like structures formed due to the programmed death of neutrophils called NETosis in response to infections or cancer [30]. Brinkmann et al. [31] for the first time reported that NETs are composed of decondensed chromatin complexed with neutrophil proteins (e.g., neutrophil elastase and myeloperoxidase) which are able to capture, neutralize, and kill microbes. NETs can contain both mtDNA and gDNA [32].

A significant increase in apoptotic signals was observed in postmortem lung sections from COVID-19 patients and the lungs of a non-human primate model infected with SARS-CoV-2 [33]. Apoptotic death of T-cells was demonstrated by André et al. [34] in severely affected COVID-19 patients. Necroptosis and pyroptosis are commonly seen in COVID-19 in conditions of low apoptosis [35]. However, the assessment of cell death in the mentioned studies is not accompanied by data on the release of cfDNA. Upcoming detailed analysis of released molecules could supplement the data on COVID-19 molecular pathology.

**Figure 1:**
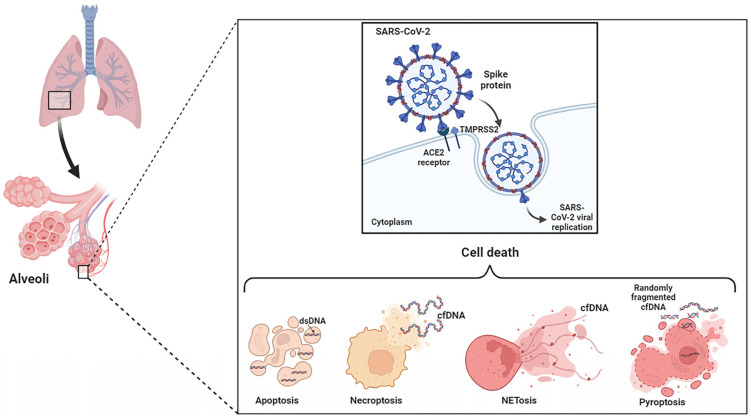
Main forms of cell death associated with COVID-19 and occurrence of cfDNA (This illustration was created with BioRender.com).

The main forms of cell death associated with COVID-19 (Figure 1) are summarized in the reviews of Rex et al. [29], Bader et al. [36], and Morais da Silva et al. [37].

#### 2.2.2. Extracellular Vesicles

EVs such as apoptotic bodies (1000–5000 nm), microvesicles (100–3000 nm), and exosomes (30–100 nm) are another source of cfDNA [38]. Thakur et al. [39] was the first to discover double-stranded gDNA (dsDNA) in exosomes from different cancer models.

Exosomes are secreted by all cell types and have been found in plasma, urine, semen, saliva, bronchial fluid, cerebrospinal fluid, breast milk, serum, amniotic fluid, synovial fluid, tears, lymph, bile, and gastric acid [38]. Whole-genome sequencing revealed that fragments of DNA in EVs (EV-DNA) can originate from any chromosome and mtDNA, which indicates a sequence-independent DNA fragment loading [40]. EVs may also contain single-stranded DNA (ssDNA) fragments [9], which can be transferred from cell to cell. Plasma cfDNA analysis in healthy donors showed that more than 93% of cfDNA is located in plasma exosomes [41]. However, cfDNA can be released into circulation through the breakdown of EVs [42].

It has been shown that cfDNA secreted in exosomes may serve to maintain tissue homeostasis by removing damaged DNA. The inhibition of exosome secretion results in the accumulation of nuclear DNA in the cytoplasm and provokes the innate immune response [43].

DNA sequences in EVs, which could be delivered from one cell to another, can regulate mRNA and protein expression, and affect the physiological function in the recipient cells [44].

#### 2.2.3. Chromosomal Instability and Micronucleus Formation

Micronuclei (MNi) have become recognized as one of the most important biomarkers of genomic instability and source of pro-inflammatory DNA in humans [45]. Mackenzie et al. [46] reported activation of cytosolic DNA sensor cyclic GMP-AMP synthase (cGAS) that triggers a type-I interferon response in micronucleated mouse embryonic fibroblasts. The authors suggest that DNA is released from MNi due to micronuclear membrane rupture, which leads to the exposure of DNA to the cGAS. Grabuschnig et al. [9] hypothesized that MNi, with their DNA cargo, may translocate to the extracellular space and serve as sources of cfDNA; however, appropriate studies supporting this hypothesis have not yet been implemented.

#### 2.2.4. Erythroblast Enucleation

An additional mechanism of active DNA release is nucleus exclusion from erythroblasts. Lam et al. [47] found that erythroid DNA comprised a significant proportion of the cfDNA pool in plasma of healthy individuals (about 30%). The fraction of erythroid derived cfDNA in plasma of anemia patients was 12.4%, which was significantly lower than that of the healthy controls. These findings are concordant with reduced erythropoietic activity in anemia patients.

### 2.3. The Main Areas of Cell-Free DNA Application in Diagnostics

The ability to recognize the size, composition, and tissue-of-origin for cfDNA has enabled the development of non-invasive diagnostic methods that are currently used to detect placenta-derived cffDNA in pregnant women, tumor-derived cfDNA in cancer patients, cfDNA derived from dead cells in transplant patients, and cfDNA derived from parasites in corresponding human infections [48]. Stawski et al. [49] highlight three main trends in cfDNA application: NIPT, oncology, and organ transplantation.

#### 2.3.1. Non-Invasive Prenatal Testing

NIPT is based on methods like NGS or other high-throughput analysis tools of free placental DNA in the serum of maternal blood to screen for fetal genetic abnormalities [50]. NIPT can be used to screen for common chromosomal aneuploidies, such as trisomy 21 (Down syndrome), trisomy 18 (Edwards syndrome), and trisomy 13 (Pätau syndrome) [51,52], as well as for other autosome and sex chromosome aneuploidies, microdeletion/microduplication syndromes [50], and for single-gene mutations [53].

NIPT allows detecting a trisomy 21 with positive predictive value (PPV) > 99%, being equal to a ~9% false positive rate; however, for unknown reasons screening for trisomies 13 and 18, is less reliable. PPVs for all other copy number changes (including sex chromosomes, other autosomes, and microdeletion/-duplications) remain substantially lower [8,54,55].

#### 2.3.2. Oncology/Liquid Biopsy

Elevated levels of circulating cfDNA in the blood of patients with various cancer types was initially demonstrated by Leon et al. [11], and now many such studies of blood-derived cfDNA are being implemented as “liquid biopsy” [56].

Rapid progress in detection methods allows determination of smaller and smaller fractions of tumor-derived cfDNA. The latter exhibits genetic and epigenetic alterations of cancers, including mutations, copy number alterations, chromosomal rearrangements, hyper-, and hypomethylation [57].

#### 2.3.3. Transplantology

Among the latest publications on the use of cfDNA as a marker of transplant rejection are the reviews of Dengu [58], Jackson et al. [59], and Reddy et al. [60]. The monitoring of the graft integrity and/or rejection can be realized by calculating the percentage of the donor-derived cfDNA in the total cfDNA (donor-derived and recipient-derived cfDNA) [59,61]. cfDNA analysis has significantly improved management of cancer patients who have undergone organ transplantation [60]. However, any otherwise induced change of cfDNA load can hinder the accuracy of the analysis [62].

### 2.4. Interim Conclusions

A brief overview and main characteristics of cfDNA are summarized in Box 1. Overall, cfDNA holds promises as a biomarker with diagnostic, prognostic, and therapeutic potential. The areas of practical application of cfDNA in the clinic are still limited, but current research in this field is very extensive and promising. No clinical applications of host cfDNA as a biomarker for viral infections are known yet. However, objective evaluation of its clinical utility remains challenging due to the lack of standardized methods for isolation, detection, and data interpretation. Moreover, the biological importance of different characteristics of cfDNA, such as tissue origin, release and clearance processes, heterogeneity, and fluctuation in pathologies, including viral infections are still not clear. At the same time, it is known that infection can result in multiple cell death outcomes, including DNA release, with both antiviral and pro-viral effects. The relationship between cfDNA and inflammatory response and blood coagulation and role of NET in triggering the immune response against pathogen invasion have been established. It was shown that EVs produced by infected cells have a distinct from healthy cells molecular signature [63]. Thus, it is expected that cfDNA assays can be considered as promising indicators of viral infections and will be more widely integrated into practice after further technological development and validation in clinical trials. The currently known data on cfDNA in viral infections will be discussed in the following sections.

Box 1 **Brief overview of main characteristics of cfDNA**

- Sources of cell-free DNA
  Apoptosis, necrosis and NETosis, extracellular vesicles (EVs), chromosomal instability and micronucleus formation, erythroblast enucleation.

- cfDNA detection methods
  PCR, NGS, and droplet digital PCR.

- The types of genetic changes identifiable
  Genetic and epigenetic alterations, including genetic mutations and copy number variations, chromosomal aneuploidies.

- Biological activities of cfDNA
  Regulation of the immune system, coagulation pathway, intercellular communication, and removal of damaged DNA.

- Application of cfDNA
  Non-invasive biomarker for prenatal testing, organ transplantation and cancer detection, monitoring, and treatment response assessment.

- Challenges and limitations
  Standardization of isolation, detection, and data interpretation methods is lacking, hindering objective evaluation of cfDNA’s clinical utility.

## 3. Cell-Free DNA in SARS-CoV-2 Infection

### 3.1. Circulating Cell-Free DNA and Its Tissue Origin

The studies presented in this section characterize circulating cfDNA in the blood (plasma or serum) with an unidentified nucleotide sequence, not encapsulated in vesicles. Another group of studies presented in the next section focuses on mitochondrial and telomeric cfDNA, which have been identified due to their specific role in inflammation.

Liu [64] hypothesized that cfDNA released from damaged cells in cytoplasm or blood can explain many symptoms of COVID-19, such as cytokine storm, acute respiratory distress syndrome (ARDS), mucus plug, and acute injuries of the heart, liver, and kidney. Hammad et al. [65] revealed a significant increase in plasma cfDNA among severe compared with non-severe COVID-19 cases. According to the authors, COVID-19-associated lymphopenia due to lymphocyte apoptosis could contribute to a greater release of cfDNA in severely affected patients. Recently, Mishra et al. [66] demonstrated predictive values based on plasma levels of cfDNA for discrimination of severe from mild course COVID-19 patients; these indicate the applicability of cfDNA for stratification and personalized monitoring of COVID-19 patients.

A significant link between antiphospholipid autoantibodies, namely anti-cardiolipin autoantibodies IgG, and serum cfDNA in patients with severe form of COVID-19 was found, that could intensify the thrombo-inflammatory state. Additionally, severely affected patients displayed higher levels of NET biomarker citrullinated histone H3 than mildly affected ones [67].

M2Model, a LightGBM-based machine learning model was developed to predict critical COVID-19 cases at admission by analyzing laboratory parameters and cfDNA profiles in plasma. Fragment length ratio, transcription start site coverage score, and frequency of 4-nucleotide motifs at the 5′ fragment of cfDNA were analyzed using WGS data. In total, 4 laboratory results and 17 transcription start sites (TSS) of 510 combined features were identified as the top-predictive features; they accounted for 37.9% of total feature importance. Remarkably, TSS coverage score features alone contributed the most towards critical COVID-19 course prediction [27].

Data on cfDNA in COVID-19 patients are summarized in Table 1.

Identification of the tissue origin of cfDNA may be important for its more accurate use as a biomarker for COVID-19. Although the lung is the predominant locus of serious SARS-CoV-2 infections, multiple organs and tissues such as hematologic, cardiovascular, renal, gastrointestinal and hepatobiliary, endocrinologic, neurologic, ophthalmologic, and dermatologic systems can be affected in corresponding patients [68]. Tracking tissue sources of cfDNA is possible based on DNA methylation patterns and their comparison with the reference human methylome atlas [69].

cfDNA methylome reflects the involvement of multiple cells/tissues/organs and thus can represent the full spectrum of possible changes in COVID-19 pathogenesis. Cheng et al. [70] revealed that cfDNA methylation profiles in COVID-19 patients were subject-dependent, and that changes in cfDNA tissue profiles occur gradually over days rather than hours. Significant increases in the relative proportion of lung-, liver-, and erythroblast-derived cfDNA was revealed in the blood of COVID-19 patients, which was likely related to COVID-19-associated tissue injury. The concentration of cfDNA correlated with the World Health Organization ordinal scale for disease progression, with an increase in cfDNA being strongly associated with admission to the intensive care unit (ICU) and need for mechanical ventilation.

The major sources of total plasma cfDNA both nuclear and mitochondrial origin in COVID-19 patients were hematopoietic cells. cfDNA was derived also from the vascular endothelium, adipocytes, liver, pancreas, intestine, bladder, kidney, heart, and lung. cfDNA levels positively correlated with COVID-19 disease severity, indicators of inflammation (C-reactive protein), and thrombosis (D-dimer) [71]. cfDNA levels in patients with influenza and respiratory syncytial virus (RSV) were significantly lower and differed in tissue origin from those in COVID-19 patients.

Chen et al. [72] presented the first study on the utility of genome-wide 5-hydroxymethylcytosine (5hmC) profiles in plasma cfDNA as an accurate early warning marker for COVID-19 progression and myocardial injury in COVID-19 patients. Among 5hmC-modified genes, Chen et al. [72] highlighted the link of phosphodiesterase 4D (PDE4D) and ten-eleven translocation 2 (TET2) with COVID-19 manifestation. *PDE4D* gene, which plays an important role in the immune signaling pathway, was highly expressed in heart tissue of COVID-19 patients. TET2 enzyme, which catalyzes the conversion of 5-methylcytosine (5mC) to 5hmC, had a lower 5hmC level in myocardial injury patients.

### 3.2. Cell-Free mtDNA

Mitochondria are among the most active players in host response to viral infection. Shang et al. [73] demonstrated that SARS-CoV-2 RNA enters mitochondria through the mitochondrial outer membrane protein Tom20, and actively replicates inside the mitochondria. This is accompanied by depolarization of the mitochondrial membrane and opening of mitochondrial permeability transition pores. Changes in mitochondrial integrity, in particular, causes the release of mtDNA into the cytosol through disrupted mitochondrial membrane [74]. MtDNA released into the cytosol or extracellular space, triggers an immune response [75]. In-depth knowledge of interactions between mitochondria and SARS-CoV-2 and other viruses is essential to develop knowledge about the mechanisms of infectious diseases. Available publications mainly deal with mtDNA in diseases associated with inflammation and some viral infections. So far, only two controversial studies address questions relating to the extracellular mtDNA as an indicator of COVID-19 infection.

#### 3.2.1. Characteristics of cf-mtDNA

MtDNA can be released into the cytoplasm or extracellular space via an active regulated process, or as a consequence of passive release [76]. Active release of mtDNA occurs through NETs [77] or EVs [78]. Passive release of mtDNA is mainly due to cell death by apoptosis or necrosis [79]. cf-mtDNA can be found in the extracellular space or encapsulated in vesicles. Recent evidence suggests that most of the cf-mtDNA may be encapsulated within EVs [78,80] or extracellular whole mitochondria [81,82].

cf-mtDNA exists in different biofluids, such as urine [83], blood [84], saliva [81], cerebrospinal [85], and follicular fluid [86]. MtDNA is supposed to be more prone to damage than nuclear DNA. This is due to the fact that protective histones are absent in the mitochondrial genome and the capabilities of the mtDNA repair system are limited [87,88]. Additionally, mtDNA is located in close proximity to the site of reactive oxygen species (ROS) production in the mitochondrial membrane, which is what makes it susceptible to damage [89]. cf-mtDNA is inducible within minutes and exhibits high inter- and intra-individual day-to-day variation, highlighting the dynamic regulation of its levels [81,90].

#### 3.2.2. cf-mtDNA-Mediated Inflammation and Related Pathologies

Sharing a remarkable number of unmethylated CpG islands with bacterial DNA [91], mtDNA when released into the extramitochondrial compartment acts as an immune trigger, called mitochondrial damage-associated molecular pattern (mtDAMP). MtDAMP molecules activate inflammatory signaling in a manner similar to pathogen-associated molecular pattern [75,92].

Release of mtDNA into the cytoplasm and out into the extracellular environment triggers a pro-inflammatory response by interacting with three important PRRs of the innate immune system: toll-like receptor 9 (TLR9), nod-like receptor (NLR) family pyrin domain containing 3 (NLRP3), and cGAS [75,93]. TLR9 and other TLRs may occur both in the cell membrane and intracellularly, and so are accessible to both cytosolic and extracellular mtDNA [93,94]. Cytosolic DNA is not extracellular, but according to Kustanovich et al. [22], it can be considered as its precursor. After the binding with unmethylated CpG islets of mtDNA, TLR9 initiates an inflammatory response via activation of MAPK and NF-kB and release of TNF-α, IL-1β, and IL-6 [95]. In addition, cytosolic mtDNA can activate NLRP3 and AIM2 (absent in melanoma 2) inflammasomes through interaction with their sensor proteins [93]. Moreover, cf-mtDNA can induce pulmonary damage in mice via activation of NLRP3 inflammasome [96], and cytosolic mtDNA can also be recognized by the cGAS. Once activated, cGAS stimulates transcription of type I interferons genes [93].

Excessive release of cf-mtDNA contributes to numerous inflammatory and autoimmune conditions, which also rises in response to viral infections.

cf-mtDNA was found to be markedly elevated in circulation and caused inflammatory responses after severe injury [97,98]. Its presence is also associated with ARDS in trauma and sepsis patients [99]. Plasma cf-mtDNA levels were significantly higher in septic shock than in sepsis patients [100]. Iske et al. [101] have shown that old mice have augmented systemic levels of cf-mtDNA, resulting in an age-specific pro-inflammatory response. Accumulation of senescent cells have been identified as a key source of cf-mtDNA.

It is expected that the level of cf-mtDNA should increase in pathologies, but there is also evidence that it may decrease. Cf-mtDNA in diabetic patients was shown to be increased in mild diabetic retinopathy and decreased in severe diabetic retinopathy with a parallel increase in mtDNA damage and inflammation [102]. The authors note that their findings describe DNA dynamics in retinopathy, but they do not offer an explanation.

Particular difficulties arise in the interpretation of cf-mtDNA levels in neurological and neuropsychiatric diseases, which are also accompanied by inflammation [103]. The contradictions found in this area reflect typical difficulties of using cf-mtDNA as a biomarker, which also occurs in other diseases. Reduced cf-mtDNA was shown in Alzheimer’s (AD) [104] and Parkinson’s disease (PD) [105] when compared to matched controls. In contrast, Sharma et al. [106] found an increase of mtDNA concentrations in serum and cerebrospinal fluid (CSF) in patients with PD compared with healthy controls, but only in women, indicating the possibility of sex-specific differences. Fernström et al. [107] explained the decrease in plasma cf-mtDNA they found in difficult-to-treat depression by concurrent psychotropic medications and co-morbidity. Recently, Park et al. [6], in their systematic review and meta-analysis of circulating cf-mtDNA in healthy and diseased brain, showed that there is a decreasing trend in CSF peripheral blood cf-mtDNA concentrations in patients with non-psychiatric neurological illnesses compared with healthy controls. However, no changes of cf-mtDNA concentrations in patients with neuropsychiatric conditions were found.

According to Gambardella et al. [108], a decreased amount of cf-mtDNA in neuroimmunological diseases is related to progressive cell dysfunction and mitochondrial loss leading to reduced mtDNA release. On the other hand, elevation of CSF cf-mtDNA levels occur during acute inflammation, which anticipates the neurodegenerative process.

#### 3.2.3. Interaction of SARS-CoV-2 with Mitochondria as a Possible Pathway for the Formation of cf-mtDNA

Like all (+) RNA viruses, SARS-CoV-2 also induces a remodeling of host cell membranes to form viral replication organelles. Recent 3D electron microscopy reconstructions of SARS-CoV-2-infected human pulmonary epithelial Calu-3 whole cells and subcellular compartments revealed ER-derived double-membrane vesicle (DMV) formation. In addition to the formation of ER-derived DMVs, SARS-CoV-2 replication alters the morphology, the number, and the function of mitochondria [109]. SARS-CoV-2 RNA genome and subgenomic RNAs were revealed to be enriched in the host mitochondria [110]. This mitochondrial residency signal was interpreted by the authors as an indicator of intracellular SARS-CoV-2 RNA traffic that may be related to DMVs formation of mitochondrial origin. Moreover, RNA and protein components of the mitochondria were shown to be captured with the SARS-CoV-2 RNA in Vero E6 and Huh7.5 cells, suggesting a close physical interaction. In addition, changes in mitochondrial shape and increase in size after infection was found using electron microscopy [111].

Results of these studies suggest that mitochondria membranes can also be used to form virus-containing double-membrane vesicles, a critical stage in the coronavirus life cycle [112,113]. Nevertheless, a potential role for mitochondria in DMVs biogenesis is, to date, based only on indirect experiments [113]. As the virus uses mitochondrial machinery to replicate, mitochondrial membranes can be damaged and mtDNA can be released into the cytosol and circulation [112,114].

#### 3.2.4. cf-mtDNA in COVID-19

cf-mtDNA levels in COVID-19 patients are reported in two publications with inconsistent results. Valdés-Aguayo et al. [115] found elevated levels of cf-mtDNA in whole blood of COVID-19 patients compared with controls. Lower concentrations of cf-mtDNA were observed in patients with severe versus mild to moderate COVID-19, and in severe course survivors versus deceased patients. The severe form of COVID-19 appears to be accompanied by mitochondrial dysfunction and decreased cf-mtDNA levels. Mean corpuscular hemoglobin, partial thromboplastin time, and systolic blood pressure correlated positively with the concentration of cf-mtDNA.

In contrast to the results of Valdés-Aguayo et al. [115], Scozzi et al. [116] reported that elevated plasma cf-mtDNA levels are associated with COVID-19 severity. COVID-19 patients with high cf-mtDNA levels are more likely to require admission to the ICU and intubation, and they have a higher risk of death. Significant correlations between indicators of necrosis lactic acid dehydrogenase (LDH) and IL-6 with cf-mtDNA levels point to a potential harmful role of cellular necrosis in COVID-19 pathophysiology. Valdés-Aguayo et al. [115] supposed that these differences between studies may be explained by the different time of blood sampling and the origin of the biological samples from which the mtDNA was quantified (plasma vs. whole blood). Nonetheless, it is obvious that release of mtDNA from the damaged or dying cells into the cytoplasm or circulation can influence immune response against COVID-19 [117].

Discussing mitochondrial dysfunction in COVID-19 patients, Saleh et al. [118] suggested that less explored extracellular mitochondria may affect blood coagulation, clot, and thrombosis formation and thereby influence COVID-19 pathogenesis. The potential role for circulating free mitochondria in the blood of COVID-19 patients remains to be elucidated.

### 3.3. Cell-Free Telomeric DNA, Progress, and Application Perspectives

#### 3.3.1. Characteristics of cf-telDNA

Telomeres are nucleoprotein structures found at the end of each chromosome arm that function to maintain genome stability. The length of telomeres is known to shorten with each cell division, and it is well established that telomere attrition is related to replicative capacity in vitro. Telomere length and telomerase activity are important players in human diseases and aging [119]. The contribution of telomere shortening to the process of age-related cardiometabolic and neurological disorders as well as tumorigenesis and cancer has been well characterized and is generally accepted [120].

Although associations between telomere length and cancer risk or prognosis have been extensively investigated in target tumor and non-tumor tissues, e.g., in liver cells [121]. Telomeres in peripheral blood leukocytes are attracting more attention for their easy accessibility. Most recently, a relationship between telomere length in leukocytes of patients with stroke [122], osteosarcopenia [123], colorectal cancer [124], schizophrenia and related disorders [125], type 2 diabetes [126], and COVID-19 infection [117] has been shown.

Circulating telomeric DNA is becoming a promising target in early disease detection, therapy response monitoring, and prognosis evaluation. Telomere shortening causes the depletion of telomeric sequences in cytoplasmic/cfDNA pool. Thus, the assessment of telomeric cfDNA in the extracellular fraction partially reflects changes in telomere length [127].

There are only a few studies that provide evaluation of telomeric sequences in extracellular DNA. A review by Gezer et al. [128] on the application of repetitive DNA elements in liquid biopsy provides examples of telomeric cfDNA (cf-telDNA) quantification.

#### 3.3.2. cf-telDNA-Mediated Inflammation and Related Pathologies

Cytoplasmic and extracellular DNA were proven to act as potent inductors of inflammation [129]; on the contrary, cf-telDNA sequences have been shown to exert anti-inflammatory activities [127,130]. CpG-containing bacterial and viral DNA (CpG-DNA) elicits immunostimulatory activity via TLRs [131]. At the same time, CpG-DNA are relatively rare in the human genome, but there are many of these sequences in telomeres. Interestingly, cf-telDNA reduces inflammation by competing for receptors with viral and bacterial DNA [127,130].

Suppressive synthetic oligodeoxynucleotides (ODNs), composed entirely of TTAGGG multimers, effectively down-regulate CpG-induced immune activation in a mouse model [132]. The suppressive activity correlates with the ability of telomeric TTAGGG repeats to form G-tetrads. A practical application of the anti-inflammatory ability of telomeric repeats is the successful design and synthesis of artificial ODNs, the best-known example being A151, composed of four TTAGGG motifs [133,134,135].

Telomeric sequences in plasma and serum cfDNA may influence the immune response in the THP1 monocytic cell line by inhibiting TNF-α mRNA expression [136]. An age-related reduction in the content of cf-telDNA is considered as a pro-inflammatory factor in inflamm-aging [127]. The presence of higher than expected telomeric DNA sequences in EVs from centenarian’s fibroblasts confirms telomeres anti-inflammatory activity [127].

Muñoz-Lorente et al. [137] established embryonic stem cells with telomeres of twice the normal size, and chimeric mice containing cells with both hyper-long and normal telomeres. Mice with hyper-long telomeres showed less metabolic aging and longer lifespans. Analyzing these results, Bonafè et al. [130] suggested that a longer and healthier life of mice with hyper-long telomeres is due not only to the higher potential for cell replication, but also to the greater anti-inflammatory potential of longer telomeres.

Most of cf-telDNA research has been done on cancer, which is also strongly associated with inflammation [114]. Wu and Tanaka [138] showed a significant decrease of cf-telDNA in the serum of breast cancer patients with no prior treatment compared to control individuals. Furthermore, cf-telDNA levels distinguished even the pre-malignant ductal carcinoma group from the healthy group. The authors speculate that tumor cells with shortened telomeres might be prone to cell death and preferentially provide cfDNA. Considering that tumor suppressor gene products including BRCA1 and BRCA2 play an important role in telomere maintenance [139], the association of the plasma cf-telDNA level in unaffected women with and without mutations in *BRCA1* or/and *BRCA2* genes was determined. The results showed that the plasma cf-telDNA level was lower in unaffected *BRCA1/2* mutation carriers than in age-matched controls [140]. Relative telomere length (RTL) in serum cfDNA was found to be significantly shorter in patients with endometrial cancer [141] and gastric cancer [142] than in healthy controls. Telomeric cfDNA alterations in telomere length/levels for various types of cancer are summarized by Holesova et al. [143]. The authors concluded that cf-telDNA remains poorly explored despite its potential suitability to become an informative genetic biomarker for many cancers.

#### 3.3.3. Length of Telomeres in Patients with COVID-19

cf-telDNA in COVID-19 patients has not been studied yet, but telomere measurement in peripheral blood cells of such patients revealed an association of shorter telomere length with more severe COVID-19 disease [115,117,144,145] and adverse outcomes [146]. Retuerto et al. [147] support previous observations on the role of TL as a risk factor for hospitalization in COVID-19, but not for in hospital complications nor persistent post-COVID-19 manifestations that occurred independently of TL.

According to Aviv [148], shorter telomeres may be responsible for slow lymphocyte proliferation in SARS-CoV-2-infected patients, which may lead to leukopenia. This hypothesis was later confirmed when an association of short telomeres with leukopenia was found in elderly COVID-19 patients [149].

Anderson et al. [150] developed a model linking ageing to COVID-19-related T-cell lymphopenia and mortality. The model showed that short TL of hematopoietic cells might increase susceptibility of older adults, and some younger individuals with inherently short TL, to COVID-19-related T-cell lymphopenia and severe disease. Haridoss et al. [151] found conflicting results on the association between shorter leukocyte telomere length and COVID-19 severity based on review of thirteen studies and meta-analysis of seven from them. Hence, additional research with age and gender adjustments are needed to draw definitive conclusions.

### 3.4. Extracellular Vesicles: Progress and Application Perspectives

EVs contain sets of genetic information available for horizontal transfer among neighbor cells by endocytosis or fusion with the cell membrane [152]. The transferred EV-DNA can penetrate into the cytosol, and even into the nuclei [153], and influence the function of the recipient cells [154].

An extracellular vesicle-associated DNA database (EV-ADD) has recently been developed [155]. Currently, it contains samples representing 23 diseases, tested in 8 different human biofluids. EV-ADD encompasses EV-DNA data representing the whole genome, oncogenes, and mtDNA.

However, more high-throughput studies are needed to establish the functional significance of EV-associated genetic material in various diseases [156]. Despite numerous studies of EVs in various pathologies, only a few of them address their DNA content. Nevertheless, EV-DNA is attracting increasing attention as a player of cell communication with potential as a clinical biomarker.

A number of studies have investigated the contribution of EVs to the progress of COVID-19.

Significant changes in the EVs found not only between healthy controls and COVID-19 patients, but also between patient subgroups demonstrate that EVs of patients reflect inflammation, thrombogenicity, and disease severity [157]. Current research is focused on the analysis changes in proteins, lipids, and miRNAs in EVs from COVID-19 patients; however, none of them analyzed the DNA content. A large-scale analysis of the composition and functions of EVs revealed temporal changes in the proteome and lipidome of EVs at different stages of COVID-19 [158]. EV-associated miRNAs that regulate mRNA transcripts and signaling pathways after uptake by recipient cells have been shown to have an important regulatory function in the immune response to SARS-CoV-2 and the progression of severe disease [159].

Su et al. [160] demonstrated increased levels of EV-associated tissue factor (TF), coatomer protein complex beta subunit (COPB2), interleukin-18 receptor 1 (IL-18R1) activated caspase 3, and domain mixed-origin kinase-like (MLKL) with risk of microthrombosis, malignant disease progression, and hospital stay in patients with COVID-19. In contrast, patients with high levels of COPB2 required for Golgi budding and vesicular transport may be more resistant to disease progression.

### 3.5. Cell-Free DNA Derived from NETs

Neutrophils can release chromatin structures coated by antibacterial proteins, called NETs to prevent pathogen spreading during infectious diseases [161]. Three markers are commonly used to detect NETs in blood: cfDNA, myeloperoxidase-DNA complex (MPO-DNA), and citrullinated histone H3 (Cit-H3). Recently, Henry et al. [162] validated cfDNA as a marker of NETosis by demonstrating its correlation with serum levels of NETs components MPO and neutrophil elastase.

Clinical and experimental data have revealed a role for NETs in COVID-19 disease [163]. The relevance and potential significance of NETosis in the pathogenesis of COVID-19 are summarized in Table 2.

SARS-CoV-2 virus-induced NETosis activation in human neutrophils in vitro was shown for the first time by Arcanjo et al. [164] using extracellular DNA measurement. cfDNA release in neutrophils was shown to be induced by the virus and also by the serum of severe COVID-19 patients. Middleton et al. [165] revealed increased NETs in autopsy lung specimens from COVID-19 patients by measuring MPO-DNA complexes and its correlation with COVID-19 severity. Many neutrophils at the stage of the early NETosis were found to be localized in the pulmonary microthrombi of patients, which confirms the contribution of NETs to COVID-19-related lung injury. Skendros et al. [166] also revealed increased plasma levels of NETs in COVID-19 patients by measuring MPO-DNA complexes. In addition, it was shown that treatment of control neutrophils with patients’ plasma generated tissue factor-bearing NETs, contributing to immunothrombosis.

The concentration of NETs, measured as MPO-DNA complexes, was shown to be augmented in plasma, tracheal aspirate, and lung autopsies tissues from COVID-19 patients [167]. SARS-CoV-2 directly stimulates neutrophils to release NETs, since NETs are not formed in culture with an inactivated virus. NETs released by SARS-CoV-2-activated neutrophils promote A549 lung epithelial cell death in vitro. The addition of recombinant human DNase (rhDNase) prevented NET-induced apoptosis to similar levels observed in untreated cells. These data suggest an essential role for the extracellular DNA in promoting the cytotoxic effects of NETs.

In the sera of hospitalized COVID-19 patients, an increase in all NET indicators (cfDNA, MPO-DNA, and Cit-H3) was found. cfDNA correlated most closely with inflammatory markers [168] and higher risk of thrombosis, in spite of previous prophylactic anticoagulation [169].

Elevated plasma levels of NET markers (cfDNA, Cit-H3, and neutrophil elastase) were associated with inflammation, dysregulated hemostasis, and endothelial injury in COVID-19 patients [170].

At admission to the ICU, levels of NETosis markers (cfDNA, extracellular histone H3, and neutrophil elastase) differed significantly between the COVID-19 and non-COVID-19 groups. In the studied group of patients, cfDNA correlated better than other markers with parameters of the disease and its evolution was predictive of disease outcome [171].

de Buhr et al. [172] showed an increased level of NET markers (cfDNA and histone-associated-DNA-fragments) in the plasma of COVID-19 patients, with a higher level in men compared to women. COVID-19 patients had elevated DNase activity compared to healthy controls. The negative correlation of DNase activity with the age of male patients may be a possible severity factor of elderly male COVID-19 patients.

Excessive NETosis and NET generation are now recognized as mediators of immunothrombosis and damage to surrounding tissues and organs following SARS-CoV-2 infection, which plays a key role in COVID-19 pathogenesis [173,174]. Reduced DNase activity and resulting defective NET clearance contributes to sustained coagulation factor XII (FXII) activation in COVID-19-associated pulmonary thrombo-inflammation [175]. Lee et al. [176] demonstrated that the NETosis factor cfDNA could be a potential therapeutic target for the treatment of SARS-CoV-2-induced sepsis. The effectiveness of recombinant DNase-1 was confirmed in a septic mouse model, as well as in blood samples collected from COVID-19 patients. Efficacy of treatment with recombinant DNase 1 (dornase alfa) was also shown in a clinical trial on limited groups of COVID-19 patients [177,178,179].

**Table 2:** The relevance and potential significance of NETosis and treatment against NETs formation in the pathogenesis of COVID-19.

Origin of NETs and/or Neutrophils	Marker of NETosis/NETs	NETs Inducing Factors and Relation to COVID-19 Pathogenesis	Potential Significance	Refs
Blood of patients with severe COVID-19	MPO and cfDNA complex	Induction with SARS-CoV-2 virus and sera from severe COVID-19 patients. Positive correlation with ROS levels in neutrophils.	Treatments against NETs formation can decrease the risk of thrombosis and may have beneficial effects for COVID-19 patients.	[164]
Blood of COVID-19 patients, autopsy lung specimens, and tracheal aspirate samples	MPO and cfDNA complex	Induction with plasma from severe COVID-19 patients, and phorbol 12-myristate 13-acetate (PMA). Positive correlation with immunothrombosis, indtubation and death.	Prediction of severity of respiratory illness in COVID-19. Treatments against NETs formation can have beneficial effects for COVID-19 patients.	[165]
Plasma of patients with moderate, severe, or critical COVID-19	MPO and cfDNA complex	Induction with platelet-rich plasma from patients with severe COVID-19. Positive correlation with thrombotic potency in COVID-19.	Targeting complement activation or/and NET formation can be beneficial against COVID-19 immunothrombosis.	[166]
Blood of patients with severe and critical COVID-19	MPO and cfDNA complex	Induction with SARS-CoV-2 virus and PMA. NETs promote lung epithelial cell death in vitro.	Inhibition of NETs represents a potential therapeutic target for COVID-19.	[167]
Serum of patients with mild and severe COVID-19	cfDNA, MPO and cfDNA complex, and Cit-H3.	Induction with sera from patients with COVID-19. Positive correlation with the requirement of mechanical ventilation.	Antineutrophil therapies may be effective for patients who are at risk for progression to respiratory failure.	[168]
Serum of patients with mild and severe COVID-19	cfDNA, MPO and cfDNA complex, and Cit-H3	Positive correlation with the risk of thrombosis and worse oxygen efficiency.	Treatment with neutrophil elastase inhibitors, peptidylarginine deiminase 4 inhibitors, and adenosine receptor agonists can be beneficial against COVID-19 immunothrombosis.	[169]
Plasma of patients with moderate and severe COVID-19	cfDNA, Cit-H3, and neutrophil elastase	Positive correlation with markers of inflammation, coagulation, fibrinolysis, dysregulated hemostasis, endothelial injury, respiratory support requirement, and short-term mortality.	Predictive marker of disease severity and clinical outcome. Supports the treatment options that are able to target NETosis.	[170]
Plasma of patients with severe COVID-19	cfDNA, extracellular histone H3, and neutrophil elastase	Positive correlation with parameters of tissue damage and requirement of mechanical ventilation.	Predictive marker of disease severity and clinical outcome. Supports the treatment options that are able to target NETosis or its associated cytotoxic and pro-inflammatory DAMPS in combination with existing therapies in the ICU.	[171]
Blood and plasma of patients with severe COVID-19	cfDNA and histone-associated-DNA-fragments	Induction with serum of COVID-19 patient and PMA. Positive correlation with markers of trombosis.	Predictive marker of disease severity and clinical outcome. Supports the treatment options that are able to target NETs.	[172]
Post-mortem lung sections and plasma of patients with COVID-19	Neutrophil elastase, Cit-H3, cfDNA and MPO, and degranulated neutrophils releasing elongated chromatin-positive NETs into the lung parenchyma and the alveolar spaces	Induction with plasma of COVID-19 patient and PMA. Positive correlation with markers of inflammation and trombosis. Decreased levels of extracellular DNases.	Predictive marker of inflammation and elevated risk of trombosis. Supports the treatment options that are able to target NETs and coagulation factor XII.	[175]
Blood and serum of patients with COVID-19	Neutrophil MPO, Cit-H3 and cfDNA	Induction with PMA. Associated with sepsis and poor outcome and decreased levels of extracellular DNase-1.	Predictive marker of sepsis and poor outcome. Supports the treatment options such as nanoparticulate DNase-1 in life-threatening SARS-CoV-2-mediated illnesses.	[176]
Patients with COVID-19 requiring ICU	-	-	Supports the treatment options such as Dornase alfa (recombinant human DNase 1).	[177]
Patients with COVID-19 requiring ICU	MPO and cfDNA complex	-	Supports the treatment options such as Dornase alfa.	[178]
Patients with COVID-19 requiring ICU	-	-	Supports the treatment options such as Dornase alfa.	[179]

### 3.6. cfDNA and COVID-19-Associated Risk Factors

The effectiveness of cfDNA as a biomarker of COVID-19 is supported by the fact that changes in cfDNA levels have been detected in pathologies associated with symptoms and complications of coronavirus infection [19]. COVID-19 risk factors such as age, obesity, and diabetes were shown to be associated with elevated levels of cfDNA [3,180]. Patients with cancer were more susceptible to COVID-19 [181]. At the same time cfDNA is considered as a marker for the early detection of cancer, accurate monitoring of disease progression, and improvement of treatment [182]. 

One of the complications of COVID-19 is sepsis [183], which is also associated with elevated levels of cfDNA [184]. Increased levels of cf-mtDNA was shown to be associated with mortality of patients in the ICUs [185] and ARDS [186], conditions common in severe COVID-19 cases. cfDNA can be detected in autoimmune disorders [5]. Autoimmunity is one of the hallmarks of patients with severe COVID-19 and symptoms of post-COVID syndrome [187]. Moreover, patients with certain autoimmune diseases are more susceptible to SARS-CoV-2 infection and conversely, SARS-CoV-2 infection can cause certain autoimmune diseases [188].

The presence of cfDNA in symptoms and pathologies associated with coronavirus infection once again confirms the relevance of cfDNA as a biomarker reflecting the pathological process.

### 3.7. Interim Conclusions

Initial data on increased levels of cfDNA and the relationship between laboratory parameters and cfDNA profiles with the course of COVID-19 have already been obtained. Andargie et al. [71] suggest that if validated, blood cfDNA will be used as a noninvasive clinical tool to identify high-risk COVID-19 patients. However, at the same time, cfDNA kinetics and reference interval must be well-evaluated [71]. Stawski et al. [19] highlight the fast kinetics of cfDNA that allows providing real-time observation of COVID-19 progress. Moreover, this indicator has often been shown to be more sensitive than clinical parameters.

Extracellular mitochondrial and telomeric DNA have a special contribution to the formation of antiviral immune responses and require particular attention. The involvement of cf-mtDNA in pro-inflammatory reactions, proven in COVID-19, makes further studies of cf-mtDNA to be considered as promising and worthy of continuation. However, it should be taken into account the dependence of cf-mtDNA levels on many disease-associated factors, as well as the need to standardize methods for the collection and processing of biological samples. Gezer et al. [128] suggested that cfDNA containing repetitive sequences, including telomeric ones, can be successfully used to develop accurate biomarkers with higher sensitivity and specificity in liquid biopsy. Considering the consistency of the above studies on the relationship between telomere length in leukocytes and manifestation of COVID-19, it would be reasonable to add circulating telomeric DNA analysis to the measurement of telomere length in future research.

Molecular profiles of circulating EVs in COVID-19 patients may provide a key to understand the progression of this disease. However, current research is based on proteomics, lipidomics, and RNA profiling. As noted in recent reviews [189,190,191], EV-DNA have not yet been sufficiently studied at all, and in viral infections in particular. There are still no published data on the content of DNA in COVID-19-derived EVs. In a recent study of DNA contents of small EVs in human blood plasma, Lichá et al. [192] recommended further focusing on the potential harmful autoimmune effect of DNA in EVs.

The effects of the SARS-CoV-2 on NETs status were studied in vitro and in vivo with the wide use of cfDNA as a biomarker. Higher levels of NETs were present in the plasma of COVID-19 patients compared to healthy controls. The results obtained demonstrate a deleterious role of NETs in pathophysiology of severe COVID-19. Anti-NETs therapy may mitigate SARS-CoV-2 infection-induced complications. Initial results on the possibility of suppression of NETs by DNase treatment need further development.

## 4. Cell-Free DNA in HIV Infection

### 4.1. Cell-Free mtDNA

cf-mtDNA research is a promising area for the development of new biomarkers for the evaluation of severity and progression of viral infections [193]. The relevance and potential significance of cf-mtDNA in HIV infections are summarized in Table 3.

mtDNA plasma levels were significantly higher in patients with acute HIV infection and late presenters than in healthy individuals or in long-term non-progressors [194]. Ali et al. [193] found a seven-fold increase in the level of cfDNA in the serum of HIV-infected individuals. In contrast, Lauring et al. [195] did not find any association between plasma mtDNA and HIV disease state; however, they noted the small sample size as a limitation of their study. cf-mtDNA was found to be elevated in untreated HIV-infected patients, and decreased after treatment. These findings demonstrate the role of cf-mtDNA to assess the effectiveness of anti-retroviral therapies in HIV-infected individuals [196].

Higher cf-mtDNA plasma levels were shown to be associated with HIV infection, elevated IL-6 levels, younger age, and higher white blood cell count [197]. However Arshad et al. [197] found 3–7 times lower levels of cf-mtDNA than reported by Cossarizza et al. [194] and Lauring et al. [195], and explains this difference by plasma purification from cells and platelets using higher speed centrifugation. cfDNA levels vary widely also in other publications because of the different protocols used for the isolation of cfDNA. Thus, standardization of the cfDNA assessment methodology is required for its use as a biomarker.

Cf-mtDNA in CSF of HIV-infected subjects was shown to be correlated with early viral rebound, inflammation, and severity of neurocognitive deficits [198]. In another group of HIV patients with therapeutically suppressed HIV RNA levels in blood, higher cf-mtDNA levels were shown to be associated with inflammation, but at the same time with better neurocognitive performance and with less neuronal damage [199]. The authors explain this discrepancy by the fact that their previous study included a heterogeneous group of HIV-infected patients both on and off therapy; most of them were not virally suppressed.

Johnston et al. [200] revealed higher levels of urine cf-mtDNA in HIV-infected older subjects with less robust physical condition. These findings suggest urine cf-mtDNA as mediator and marker of inflammatory dysregulation may reflect pathophysiologic aging processes in HIV-infected patients.

**Table 3:** Relevance of cf-mtDNA levels in HIV infection.

Changes in cf-mtDNA Level	Methods of cfDNA Analysis	Potential Significance	Refs
Elevated in plasma of acute HIV patients and late presenters.	qPCR	Indication of virus-induced cell damage and inflammation.	[194]
No difference between controls and HIV patients. Negative correlation with age in HIV patients.	qPCR	A biomarker for age-related changes.	[195]
Elevated in serum of untreated HIV patients.	qPCR	A biomarker of disease progression and efectiveness of anti-retroviral treatment.	[196]
Elevated in serum of HIV patients.	qPCR	A biomarker of increased inflammation.	[193]
Elevated in plasma of HIV patients. Negative correlation with age in HIV patients. Positive correlation with levels of IL-6 and WBC in HIV patients.	Monochromatic multiplex qPCR	Might be involved in HIV-associated inflammation and a biomarker of cell death.	[197]
Positive correlation between CSF cf-mtDNA with worse learning and motor functioning, IP-10 and MCP-1, and severity of neurocognitive dysfunction.	Droplet digital PCR	A biomarker of increased inflammation in patients with HIV and HIV-related neurocognitive dysfunction.	[199]
Elevated in subjects with with a previous diagnosis of AIDS. Positive correlation between CSF cf-mtDNA with better neurocognitive performance, MCP-1, TNF-α, IL-8. Negative correlation with CD4+ T-cell nadir.	Droplet digital PCR	A biomarker of increased inflammation regardless of detectable viral replication, and HIV-related neurocognitive dysfunction.	[199]
Negative correlation between levels of urine cf-mtDNA in HIV-infected older subjects with robust physical condition. Positive correlation with TNF-α.	qPCR	A biomarker of of pathophysiologic aging processes that predispose older adults with HIV to geriatric syndromes.	[200]

### 4.2. Extracellular Vesicles

Data obtained in different virus-mediated diseases, including HIV, indicate that EV-based mechanisms can impact viral pathogenicity, facilitate viral spread, and modulate antiviral immune responses [63,193,194,201,202]. Lee [203] provides a comprehensive summary of the potential roles of Evs in HIV-1 infection. Evs participate in the interplay between virus and host and play both pro- and antiviral roles. So far, it is not known how these diverse functions of Evs can be explained. Therefore, further studies of HIV-induced EVs, including their DNA cargo as well as improvement of methodological approaches, should be implemented.

### 4.3. Neutrophil Extracellular Traps

NETs represent a multilayered defense against viral infection, sequestering viral particles, preventing fusion of virus with target cells and direct neutralization of virions [204]. NETs production in HIV infection and viral immune evasion mechanisms targeting NETs are presented, in particular, by Saitoh et al. [205] and Mojoli et al. [206], and summarized in reviews of Jenne and Kubes [204], Schönrich and Raftery [207], and Schultz et al. [161]. The addition of NETs, initially treated with DNase I, to HIV-1-infected macrophages significantly diminished the anti-HIV-1 effect of NETs [206], so DNase treatment impairs the ability of the NETs to suppress HIV infection.

## 5. Cell-Free DNA in Hepatitis Virus Infection

### 5.1. Cell-Free mtDNA

According to Ali et al. [193], mtDNA is a promising area for the development of markers of viral infections, but it still needs exploration. Studies indicating the relevance and potential significance of cf-mtDNA in hepatitis virus infection are summarized in Table 4.

A lower cf-mtDNA content in serum samples from HBV-related hepatocellular carcinoma (HCC) patients and cancer-free HBV controls was shown to be significantly associated with an increased risk of HCC in HBV-infected patients [208]. Comparing viral infections, Ali et al. [193] found that hepatitis C (HCV) patients have the highest levels of cf-mtDNA, followed by HBV and HIV patients. A significant correlation was found between cf-mtDNA and IL-6 only among HBV patients. Viral load was not significantly correlated with cf-mtDNA in any of the studied infections. These data need further interpretation, but indicate cfDNA as a biomarker is specific for various infections.

### 5.2. Cell-Free telDNA

Longer RTL in serum cfDNA was associated with an increased risk of HBV-related non-cirrhotic HCC [209], while Wan et al. [210] showed that longer RTL in serum cfDNA was associated with a significantly increased risk of HBV-related cirrhosis. The results of Fu et al. [209] and Wan et al. [210] were obtained by retrospective analysis; subsequent prospective and longitudinal evaluation confirmed the increased risk of hepatocellular carcinoma in HBV patients with long RTL [211].

### 5.3. Extracellular Vesicles

The involvement of EVs in hepatitis viral infections is considered in reviews of Martins et al. [63], Caobi et al. [201], Bello-Morales et al. [202], Giannessi et al. [212], and Kutchy et al. [213]. No known publications have examined the presence of host DNA in EVs, as it was with other viral infections.

### 5.4. Neutrophil Extracellular Traps

Recent studies have examined the role of NET release in the response to hepatitis viral infections. With the use of a cf-DNA/NETs marker, it was shown that HBV may inhibit NET release to escape the immune system and promote the establishment of chronic infection [214]. Analysis of the role and mechanism of NETs in the pathogenesis of viral hepatitis was carried out using MPO–DNA level, a recognized biomarker of NETs in circulation [215]. Increased hepatic NETs formation was observed in hepatitis virus-infected mice and increased plasma NETs level was shown in patients with HBV acute liver injury. Elevated NETs levels, measured on the base of NETs-DNA concentration, were detected in HBV-positive hepatocellular carcinoma patients [216].

### 5.5. Interim Conclusions

Studies of extracellular DNA in viral infections, as well as in diseases provoked by viruses, are rather limited. The most frequently studied include cf-mtDNA and NETs, whose role in antiviral immune response is well known. However cf-mtDNA studies provide evidence of not only increased but also decreased cf-mtDNA levels in viral diseases, thus, this area requires further development.

## 6. Conclusions and Future Perspectives

cfDNA is already used in prenatal diagnostics, transplantology, and oncology as a promising, minimally invasive biomarker that correlates with various pathological conditions. Nevertheless, there is a lack of standardization in methodologies of cfDNA isolation, detection, and characteristics that should be utilized in clinical practice.

COVID-19 is associated with wide variation in individual sensitivity, severity, and long-term complications which necessitates the development of sensitive biomarkers for a personalized approach. In COVID-19 patients, associations between cfDNA and various biochemical markers of inflammation, thrombosis, and disease severity have been identified. Based on DNA methylation profiling (5hmC) in COVID-19 patients, the tissue origin of cfDNA is possible to identify, providing valuable insights into disease progression.

The possibility of evaluating the effect of vaccination against COVID-19 and post-COVID-19 complications was studied using methylation-based cfDNA biomarkers. cfDNA released from dying immune cells to blood after SARS-CoV-2 vaccination have been studied by Fox-Fisher et al. [217]. The primary vaccination caused elevated levels of B-cell cfDNA, which correlates with memory B-cells and neutralizing antibodies against SARS-CoV-2, both measured after the booster. The findings suggest that the quality of the immune response to the vaccine can be predicted by measurements taken shortly after the primary vaccination. Gerhards et al. [218] evaluated cfDNA concentrations as an indicator for tissue damage in post-COVID-syndrome patients; however, it was found to be ineffective.

It must be noted that different forms of cell death are major sources of cfDNA. However, studies of the main forms of cell death in COVID-19 are not accompanied by an assessment of cfDNA release, yet. DNA content of EVs in COVID-19 and other viral infections has also not been investigated.

Since cfDNA research in COVID-19 is limited, we also had a closer look on other viral infections, inflammation-related pathologies, and COVID-19-associated risk factors to better understand the current state and perspectives in this area.

A review of the current literature shows alterations in the levels of non-vesicular host cfDNA in different viral infections such as HIV, influenza virus, HCV, and HBV viruses. The diagnostic, prognostic, and therapeutic importance of NETs, as the most frequently studied type of cfDNA in viral infections, is demonstrated. Moreover, targeting cfDNA of NETs can be suggested as a promising new approach for the treatment of life-threatening complications induced by SARS-CoV-2 infection.

In recent publications, special attention is paid to the identification of telomeric and mitochondrial sequences in cfDNA because of their association with inflammation. Inflammation plays a key role in the development and progression of viral diseases. Few publications describe cf-mtDNA levels in various viral infections, and two of them provide contradictory data on cf-mtDNA levels in COVID-19 patients. There are limited literature data concerning cf-telDNA in viral diseases, and specifically, cf-telDNA remains unexplored in patients with COVID-19.

Overall, accumulating evidence demonstrates the potential utility of cfDNA as a diagnostic and predictive biomarker and a therapeutic target in viral infections, as well. However, further research is needed to understand its role in disease progression and clinical utility.

Algorithms have been developed to predict COVID-19 severity based on routine clinical features [219] or laboratory markers and age [220]. There are rare examples of including genetic markers in such algorithms. Li et al. [221], along with general clinical indicators, took into consideration alternative lengthening of telomeres (ALT). ALT ranked in the top 10 variables with the highest importance. A machine learning model was developed based on laboratory parameters and cfDNA profiles in plasma [27]. Further inclusion of cfDNA in COVID-19 predictive systems would be quite promising.

## Figures and Tables

**Table 1:** Circulating cfDNA levels in COVID-19 patients.

Disease Severity	cfDNA Levels	Methods of cfDNA Analysis	Correlation of cfDNA Level with Clinical Parameters	Potential Significance	Refs
Non-severe and severe	Elevated in plasma of severe compared to non-severe group	qPCR	Positive correlations with NLR, ferritin and C-reactive protein. Inverse correlation with T-cell percentage	Prediction of COVID-19 severity and stratification of patients	[65]
Mild and severe	Elevated in plasma of severe and in non-survivor groups compared to mild group	qPCR	Positive correlations with NLR, ferritin, lactate dehydrogenase, procalcitonin, and IL-6	Prediction of COVID-19 severity and stratification of patients	[66]
Mild and severe	Elevated in plasma of severe group	Fluorometric assay	Positive correlation with anti-cardiolipin IgG level	Prediction of exacerbated inflammatory reaction and severity of COVID-19, and development of original therapeutic strategy	[67]
Non-critical and critical	Elevated in plasma of critical group	WGS data meta-analysis	Positive correlation with lactate dehydrogenase, prealbumin, uric acid, and α-hydroxybutyrate dehydrogenase	Prediction of COVID-19 severity	[27]

**Table 4:** Relevance of cf-mtDNA levels in hepatitis virus infection.

Virus	Disease	Changes in cf-mtDNA Level	Methods of cfDNA Analysis	Potential Significance	Refs
HBV	HBV-related hepatocellular carcinoma.	Decreased in HBV patients with HCC. Negative correlation with age in HBV male patients without HCC.	qPCR	A biomarker of HCC risk in HBV patients.	[208]
HCV	HCV-related hepatocellular carcinoma.	Elevated in serum of HCV patients.	qPCR	A biomarker of increased inflammation in patients with HCV, HBV, and HIV.	[196]
HBV	HBV-related hepatocellular carcinoma	Elevated in serum of HBV patients. Positive correlation with the level of IL-6.	qPCR	A biomarker of increased inflammation in patients with HCV, HBV, and HIV.	[196]

## Data Availability

Not applicable.
